# Extracranial Spread of Meningiomas: Molecular Determinants, Diagnostic Pathways, and Lessons From Three Thoracic Metastases

**DOI:** 10.7759/cureus.99420

**Published:** 2025-12-16

**Authors:** Victor A Perez-Gutierrez, Muneeb Ahmed, Gopal Krishna B, Kari Hird, Michael Balatico, Muhammad Atif Waqar, Jefferson Chambers, Sikandar Ansari

**Affiliations:** 1 Department of Pulmonary and Critical Care Medicine, University of Utah School of Medicine, Salt Lake City, USA; 2 Department of Medicine, Aga Khan University Hospital, Karachi, PAK; 3 Department of Medicine, Mysore Medical College and Research Institute, Mysuru, IND; 4 Department of Pathology, University of Utah School of Medicine, Salt Lake City, USA; 5 Department of Oncology, Section of Palliative Medicine, Aga Khan University Hospital, Karachi, PAK; 6 Department of Pulmonary and Critical Care Medicine, Huntsman Cancer Institute, Salt Lake City, USA

**Keywords:** anaplastic meningioma, bap1 mutation, extra-cranial meningioma, lung metastasis, meningioma histopathology, nf2, robotic bronchoscopy

## Abstract

Meningiomas are the most common primary intracranial neoplasms of all brain tumors. Although the majority are benign, a subset displays aggressive behavior characterized by recurrence, anaplastic transformation, or, rarely, extracranial metastasis. Lung parenchyma represents the most frequent site of spread, followed by mediastinal and pleural involvement. Despite recent advances in molecular characterization, predicting metastatic potential remains challenging. We report three cases of meningiomas with thoracic metastases illustrating distinct biological trajectories. Case 1 involved a 33-year-old woman with a germline *BAP1* mutation and recurrent cerebellopontine angle meningioma (WHO grade 1). After multiple resections and radiotherapy, surveillance imaging revealed pulmonary and hilar lesions. Both EBUS-TBNA and percutaneous lung biopsy confirmed a metastatic meningioma (EMA+, SSTR2+, PR rare+, BAP1 loss). Case 2 describes a 45-year-old man with a de novo anaplastic meningioma (WHO grade 3) who later developed multiple bilateral pulmonary nodules confirmed via robotic-assisted bronchoscopy (EMA+, SSTR2+, CAM5.2-). Case 3 details a 79-year-old man with a recurrent spheno-orbital anaplastic meningioma who developed bilateral pulmonary metastases diagnosed by Ion robotic bronchoscopy and confirmed by neuropathology (SSTR2+, EMA focal+, PR+). Meningioma metastasis remains exceedingly rare, but risk increases with higher grade, recurrence, and molecular alterations such as *BAP1* loss or *NF2* inactivation. Latency varies from months in anaplastic cases to decades in low-grade tumors. Minimally invasive techniques, including EBUS-TBNA and robotic bronchoscopy, provide accurate and safe diagnostic confirmation, especially when thoracic disease is suspected. Thoracic metastases from meningioma demonstrate diverse clinical courses independent of histologic grade. Molecular profiling, particularly *BAP1* and *NF2* status, may refine risk assessment and justify risk-adapted surveillance incorporating chest imaging or PET/CT. Treatment is largely palliative, with local resection or radiotherapy offering control in selected cases. Continued integration of molecular diagnostics and modern bronchoscopic technologies may improve recognition and management of these rare metastatic events.

## Introduction

Meningiomas constitute the most common primary intracranial neoplasms, accounting for approximately 13-26 % of all primary brain tumors. Typically derived from arachnoid cap cells or dural border cells, most of these tumors are benign (WHO grade 1), slow-growing, and managed with surgery and, sometimes, adjuvant radiotherapy. Although most meningiomas follow an indolent course, a small subset demonstrates aggressive features such as local invasion, recurrence, malignant histologic transformation (WHO grade 2 and 3) or, rarely, extracranial metastasis [1]. When metastasis occurs, the lung is the most predominant site, followed by bone, liver, and lymph nodes. A recent systematic review of 155 cases found that 61% of metastatic meningiomas involved the lungs [2]. The median latency to metastasis correlates with primary tumor grade: grade 3 metastases tend to appear much sooner than grade 1 tumors [2,3]. Risk factors include higher WHO grade (2 or 3), prior craniotomy, bone invasion or sinus invasion, skull-base location, and multiple intracranial recurrences. Molecular alterations (such as BAP1 loss, NF2 mutation, TERT promoter mutation, CDKN2A/B deletion) are emerging as additional markers of high-risk biology but are not yet uniformly used in clinical screening [4,5]. BAP1 (BRCA1-associated protein 1) is a tumor-suppressor gene whose loss has been associated with aggressive clinical behavior and increased metastatic potential in meningiomas. NF2 encodes the protein Merlin, and its inactivation is the most common early driver event in sporadic meningiomas, frequently associated with cytogenetic instability and higher recurrence risk. Because metastasis is rare, there are no standardized guidelines for screening, imaging, or systemic therapy. In this context, we present three illustrative cases of meningiomas with lung metastases, each exhibiting distinct biological and clinical characteristics: a case with a known germline BAP1 mutation and a long history of recurrent meningioma; a case with a de novo anaplastic (WHO grade 3) meningioma; and a case with a longstanding skull-base anaplastic meningioma complicated by severe local treatment morbidity. This highlights the importance of vigilant diagnostic measures and multidisciplinary management in these rare yet devastating situations. This case series aims to describe thoracic metastatic meningiomas, their clinical presentations, diagnostic pathways, and histologic and molecular features and to contextualize these findings within current evidence to inform risk awareness, surveillance considerations, and clinical decision-making.

## Case presentation

Case 1 

**Figure 1:**
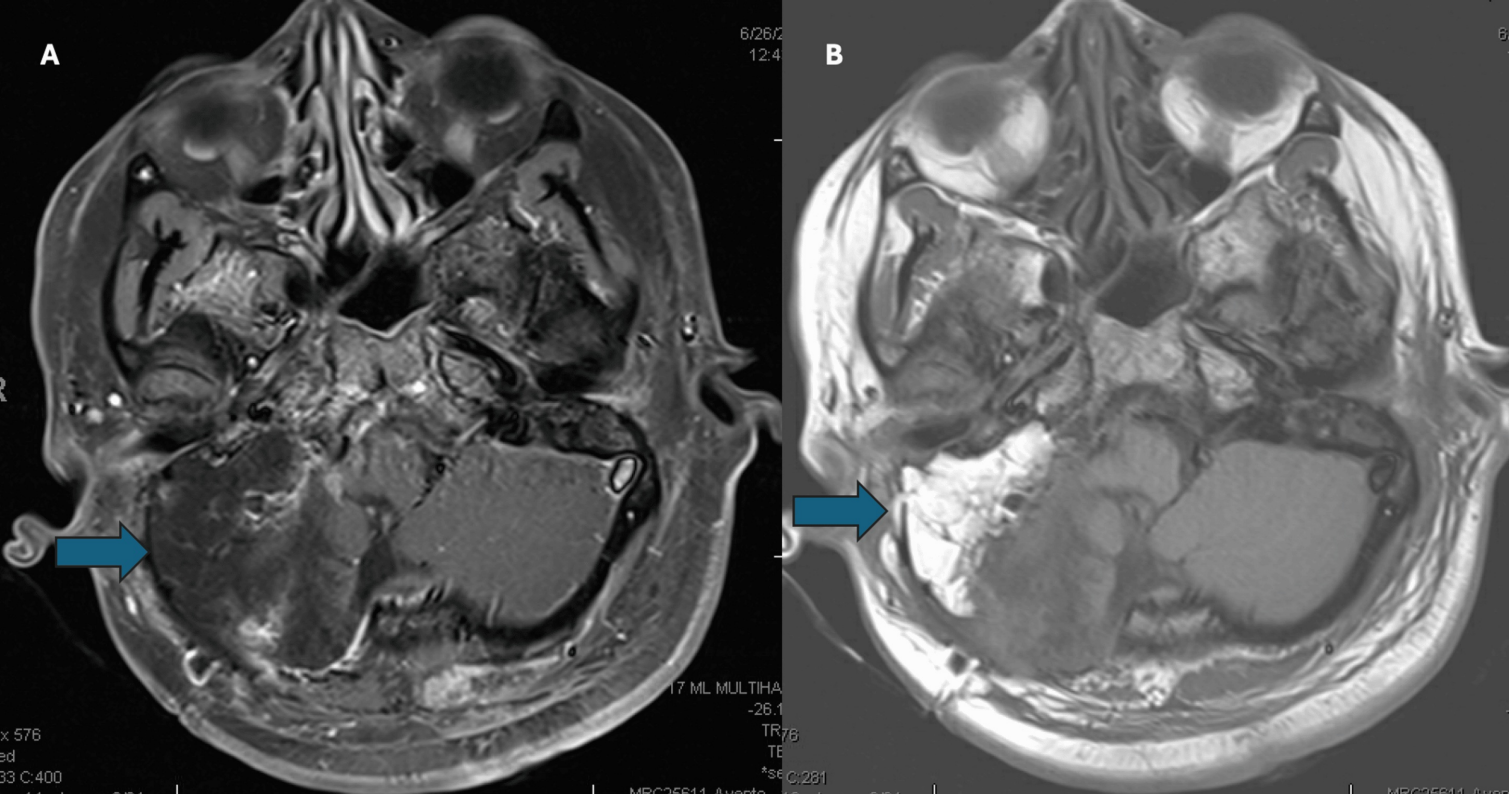
Axial brain MRI demonstrating recurrent cerebellopontine-angle meningioma in a patient with germline BAP1 mutation. (A) Axial post-contrast T1-weighted MRI showing a heterogeneously enhancing mass centered in the right cerebellopontine angle (blue arrow), with extension into the jugular foramen and hypoglossal canal, consistent with recurrent skull-base meningioma. (B) Follow-up axial post-contrast T1-weighted MRI demonstrating interval enlargement of the same lesion (blue arrow) with progressive involvement of adjacent skull-base structures despite prior resections and radiotherapy, reflecting the aggressive behavior associated with BAP1-deficient meningioma.

**Figure 2:**
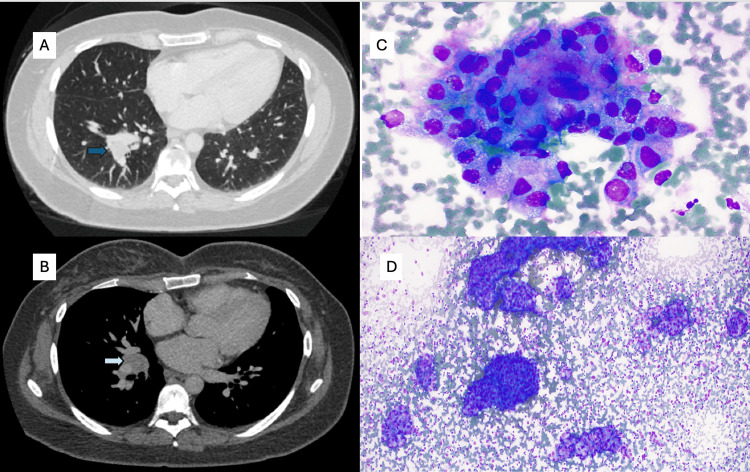
Thoracic and cytologic findings of metastatic meningioma. (A) Axial chest CT demonstrating a right lower-lobe pulmonary nodule (blue arrow) corresponding to a biopsy-proven metastatic meningioma. (B) Contrast-enhanced chest CT showing bulky right hilar and mediastinal lymphadenopathy (light blue arrow). (C) Rapid on-site evaluation (ROSE) smear (Diff-Quik, ×40) reveals cohesive clusters of epithelioid lesional cells with wispy, feathery cytoplasm and indistinct cell borders forming subtle whorled architecture. (D) Hypercellular cytology specimen (Diff-Quik, ×10) showing abundant lesional cells with cohesive, partially whirling arrangements within a background of lymphoid tissue, consistent with metastatic meningioma.

A 33-year-old woman with a known germline BAP1 mutation had a longstanding history of a recurrent right cerebellopontine angle meningioma (Figures 1A, 1B). Over more than a decade, she underwent multiple resections for disease recurrence, with histologic features varying from atypical and rhabdoid to clear cell morphology, although the most recent intracranial specimen had been classified as WHO grade 1. The tumor repeatedly involved the jugular foramen, hypoglossal canal, clivus, carotid space, and dural surfaces of the pontomedullary junction and cerebellopontine angle. Because of aggressive behavior, she received adjuvant radiotherapy, and later proton re-irradiation after marginal recurrence. Treatment complications included right-sided deafness and cranial-nerve deficits, though she remained neurologically stable. The patient was asymptomatic at the time of thoracic evaluation, and lung nodules were discovered through surveillance imaging. Chest CT scan imaging demonstrated a 1-cm right lower-lobe lung nodule and bulky right hilar lymphadenopathy (Figures 2A, 2B). Both endobronchial ultrasound-guided transbronchial needle aspiration (EBUS-TBNA) and percutaneous lung biopsy were performed. Cytology and core biopsy specimens showed atypical epithelioid cells forming nests and whorls with nuclear pseudoinclusions. Immunohistochemistry (IHC) showed EMA+, SSTR2+, PR (rare)+, and BAP1 loss, negative for CK7, CK20, TTF-1, p40, synaptophysin, S100, SOX10, lysozyme, GATA3, CD1a, and Langerin (Figures 2C, 2D). The findings, reviewed with neuropathology, were diagnostic of a metastatic meningioma. Given the low-grade histology of the most recent intracranial specimen, methylation profiling and NF2/22q sequencing were recommended to clarify molecular progression further but were not performed. She remained free of intracranial progression at the last follow-up. 

Case 2

**Figure 3:**
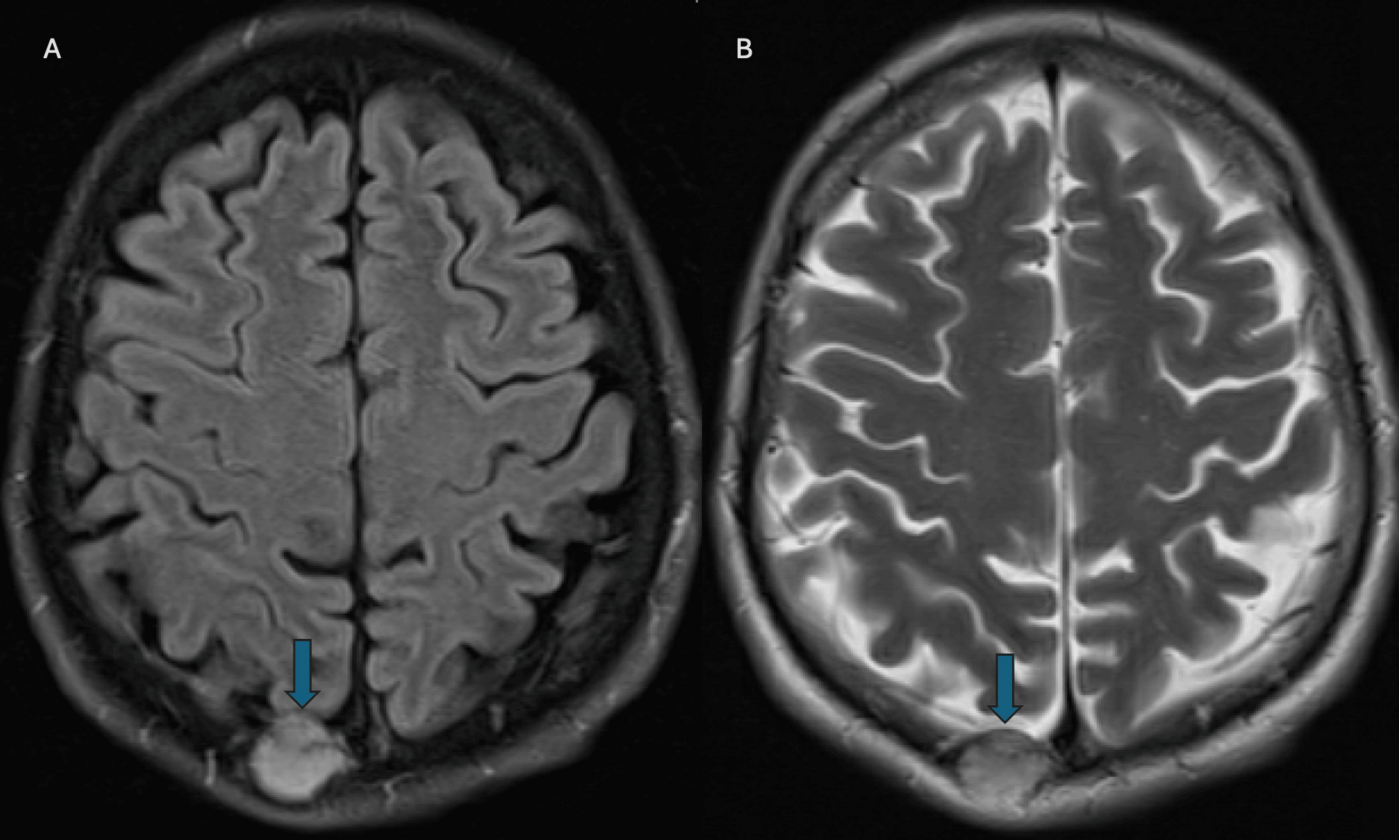
Axial brain MRI demonstrating the primary parietal calvarial meningioma. (A) Axial FLAIR MRI showing a well-circumscribed right parasagittal calvarial mass (arrow) with associated dural involvement and surrounding cortical distortion. (B) Corresponding axial T2-weighted MRI again demonstrating the extra-axial lesion (arrow), which exhibits intermediate T2 signal intensity and a broad dural base, features typical of a meningioma. The lesion abuts the superior sagittal sinus without definite invasion. FLAIR: Fluid-Attenuated Inversion Recovery

**Figure 4:**
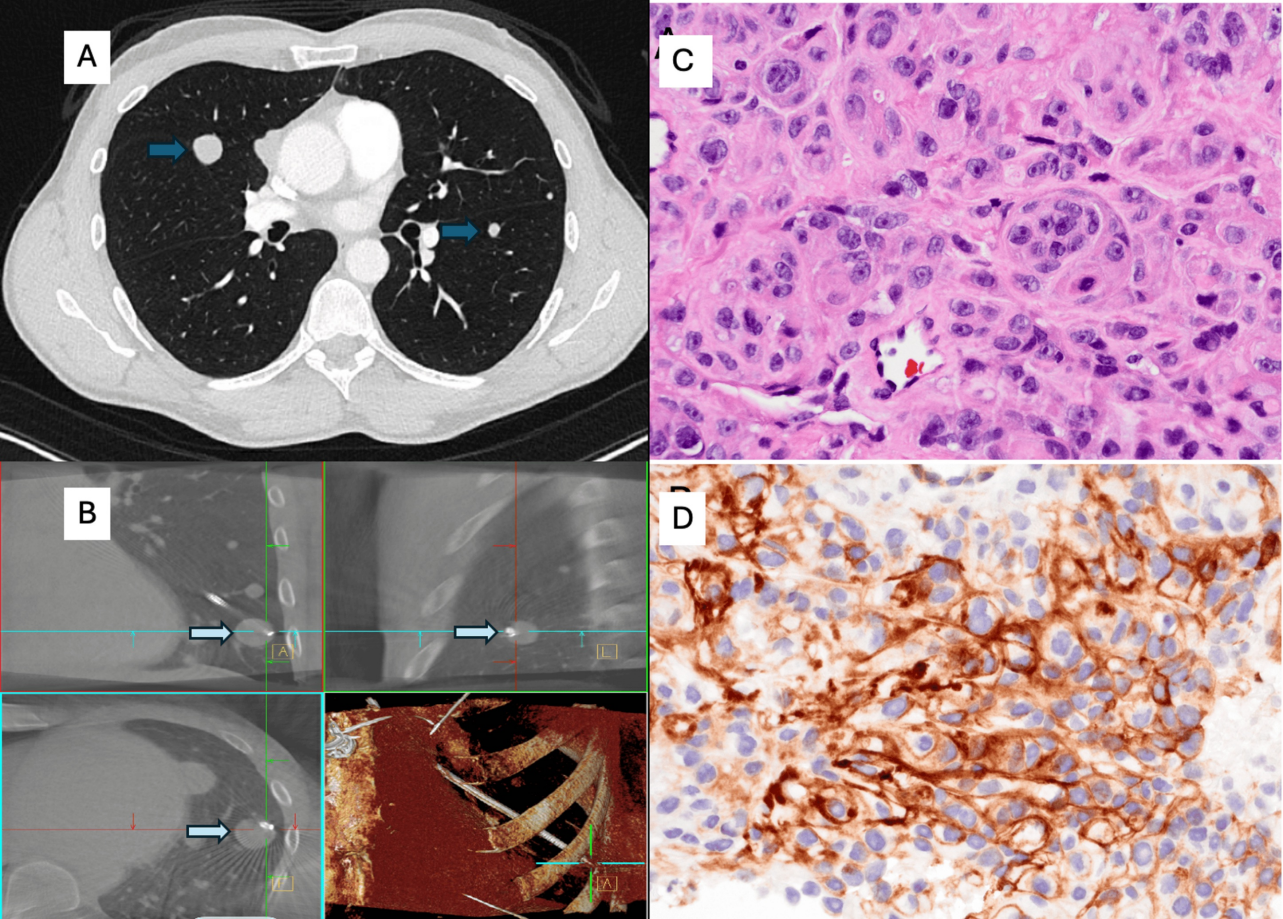
Radiologic, procedural, and histopathologic findings of metastatic anaplastic meningioma to the lung. (A) Axial chest CT demonstrating a well-circumscribed pulmonary nodule (blue arrows), subsequently confirmed as metastatic meningioma. (B) Robotic-assisted bronchoscopy with cone-beam CT demonstrating precise tool-in-lesion confirmation during transbronchial biopsy of the target left lower lobe nodule (light blue arrow). (C) Hematoxylin and eosin-stained core biopsy section showing nests and whorls of syncytial meningothelial cells with round to ovoid nuclei, prominent nucleoli, and moderate cytoplasm, characteristic of metastatic meningioma (×400). (D) Immunohistochemical staining for SSTR2A demonstrating strong membranous positivity in lesional cells, supporting meningothelial differentiation (×400).

A 45-year-old man presented with a right parietal calvarial mass incidentally discovered after noticing a firm bump on his skull. MRI of the brain revealed an avidly enhancing calvarial lesion abutting the superior sagittal sinus without invasion (Figures 3A, 3B). He underwent craniectomy with titanium-mesh cranioplasty, and histopathology demonstrated anaplastic meningioma (WHO grade 3) characterized by mitotic index up to 21 per 10 HPF, Ki-67 proliferation index up to 25%, necrosis, sheet-like growth, small-cell change, and prominent nucleoli. IHC were SSTR2+, EMA+, and STAT6-. While the tumor focally invaded bone, there was no brain invasion. Postoperative recovery was uneventful. Surveillance MRI showed no intracranial recurrence. The patient developed a mild chronic cough as pulmonary nodules evolved. Chest imaging demonstrated numerous bilateral, well-circumscribed pulmonary nodules measuring up to 2 cm, consistent with hematogenous metastases (Figure 4A). Whole-body PET/CT demonstrated avid pulmonary nodules without evidence of extrapulmonary disease. Robotic-assisted bronchoscopy with 3D navigation and tool-in-lesion confirmation was used to sample the left lower-lobe target; transbronchial needle biopsies were obtained under fluoroscopy (Figure 4B). Cytology showed epithelioid cells with abundant vacuolated cytoplasm and whorled architecture, mirroring the intracranial specimen. IHC: EMA+, SSTR2+, CAM5.2−; morphology mirrored the CNS specimen, confirming metastatic meningioma (Figures 4C, 4D). Further spinal and cranial imaging showed no leptomeningeal spread, but pulmonary burden progressed. The patient remained neurologically intact with intermittent cough and chest tightness. His case was reviewed in a multidisciplinary tumor board, and systemic treatment options were discussed in light of the poor prognosis of metastatic anaplastic meningioma. 

Case 3 

**Figure 5:**
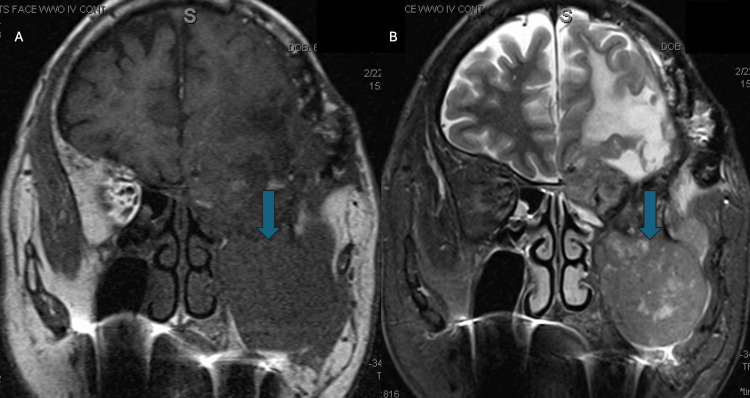
Skull-base anaplastic meningioma. (A) Coronal post-contrast T1-weighted MRI demonstrating a large, heterogeneously enhancing left spheno-orbital mass (blue arrow) with extension into the orbit, skull base, and adjacent soft tissues (blue arrow). The tumor shows irregular margins and avid enhancement, consistent with high-grade meningioma. (B) Corresponding coronal T2-weighted MRI showing the same lesion (blue arrow), which appears markedly heterogeneous with mixed solid components and surrounding mass effect. T2 signal variation reflects tumor cellularity, necrotic change, and involvement of adjacent structures including the orbit and masticator space.

**Figure 6:**
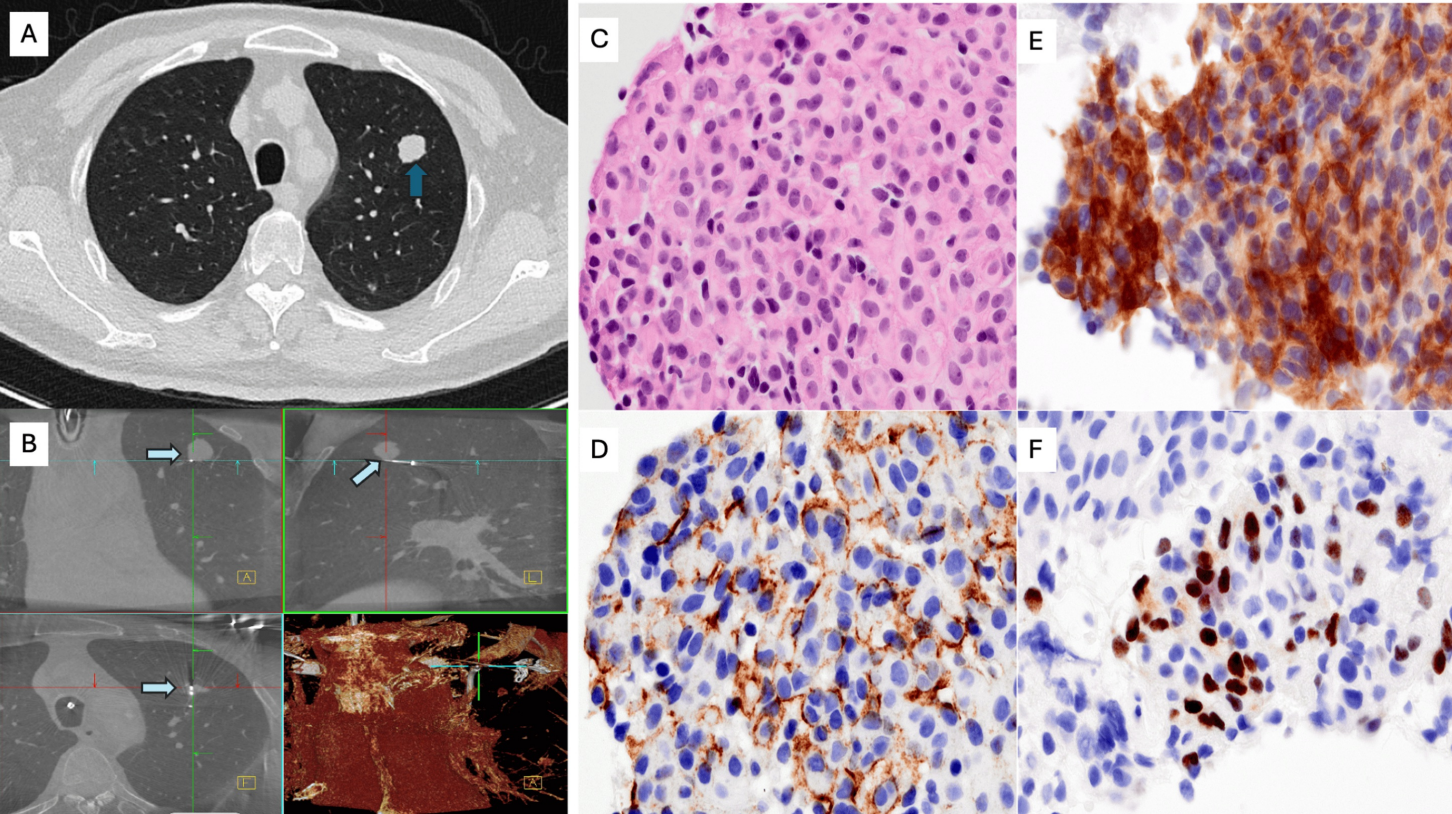
Thoracic imaging, procedural navigation, and histopathologic features of metastatic anaplastic meningioma (Case 3). A) Axial chest CT demonstrating a well-defined left upper-lobe pulmonary nodule (blue arrow), later confirmed as metastatic meningioma. (B) Ion robotic-assisted bronchoscopy with cone-beam CT showing accurate tool-in-lesion positioning during transbronchial needle sampling of the target pulmonary nodule (light blue arrow). (C) Hematoxylin and eosin-stained core biopsy section showing syncytial lobules of meningothelial cells with round to ovoid nuclei, powdery chromatin, and occasional nuclear pseudoinclusions (×400). (D) Immunohistochemical staining for SSTR2A demonstrating strong, diffuse membranous expression in lesional tumor cells (×400). (E) EMA immunostain showing robust cytoplasmic and membranous positivity in meningothelial tumor clusters (×400). (F) Progesterone receptor (PR) immunostain highlighting strong nuclear labeling in a subset of tumor cells (×400), supporting meningothelial differentiation.

A 79-year-old man with recurrent left spheno-orbital meningioma presented with progressive craniofacial disease (Figures 5A, 5B). Over several years, he underwent multiple skull-base resections and reconstructions, including frontotemporal craniotomy with transfacial skull-base resection and left orbital exenteration. Pathology revealed anaplastic meningioma (WHO grade 3) with a high mitotic index up to 25 per 10 HPF, a Ki-67 proliferation index 50%, necrosis, sheet-like growth, prominent nucleoli, and cortical-bone invasion. He completed adjuvant radiation therapy (5610 cGy in 33 fractions). Despite repeated surgical and reconstructive procedures, including cranioplasty revisions and free-flap repairs, the tumor recurred with invasion into the orbit and oral cavity. Wound infections and reconstructive flap failures complicated his postoperative course. The patient experienced progressive dysphagia, aspiration events, and declining oral intake, with pulmonary nodules identified during symptomatic evaluation. Chest imaging later revealed multiple bilateral pulmonary nodules, the largest measuring 2 cm in the left upper lobe (Figure 6A). Ion robotic bronchoscopy with cone-beam CT and 3D reconstruction confirmed precise targeting (Figure 6B). Eight 21-gauge transbronchial needle biopsies were obtained under fluoroscopic guidance without complications. Histology showed epithelioid-to-plasmacytoid cells forming syncytial whorls with meningothelial morphology. IHC: SSTR2+, EMA (focal)+, PR+, p40−, TTF-1−, synaptophysin−, consistent with metastatic meningioma (Figures 6C-6F). The diagnosis was confirmed by neuropathology review, which correlated with prior CNS specimens. Following diagnosis, his craniofacial disease continued to progress, with severe oropharyngeal dysphagia, aspiration, and malnutrition. After multidisciplinary discussion involving oncology, infectious disease, and palliative care teams, he elected for comfort-focused management and home hospice. 

A summary of the cases is presented in Table 1. 

**Table 1:** Summary of three cases of meningiomas with pulmonary or mediastinal metastases EBUS-TBNA: endobronchial ultrasound-guided transbronchial needle aspiration; RT: radiotherapy; IHC: immunohistochemistry; fx: fractions; HPF: high-power field; CNS: central nervous system

Case	Age/Sex	Primary Tumor Site & WHO Grade	Histopathologic/Molecular Features	Latency for Metastasis	Thoracic Site(s)	Diagnostic Procedure	Key IHC Findings (Metastatic Lesion)	Management/Outcome
Case 1	33 / F	Right cerebellopontine angle meningioma, WHO 1 (recurrent)	Atypical, rhabdoid, clear-cell areas; germline BAP1 mutation; BAP1 loss by IHC	16 years (first diagnosis to lung metastasis)	Right lower-lobe nodule + right hilar lymph node	EBUS-TBNA + transbronchial lung biopsy	EMA+, SSTR2+, PR rare+, BAP1 loss; CK7/CK20/TTF-1/p40/S100 negative	Palliative management; neurologically stable; no intracranial progression at last follow-up
Case 2	45 / M	Right parietal calvarial meningioma, WHO 3 (anaplastic)	Mitotic index 21/10 HPF; Ki-67 25%; necrosis; sheet-like growth; small-cell change; EMA+/SSTR2+/STAT6–; NGS and NIH methylation profiling ordered (no result)	Approx. 1 year	Multiple bilateral pulmonary nodules (≤2 cm)	Robotic-assisted bronchoscopy with 3-D navigation, fluoroscopic TBNA	EMA+, SSTR2+, CAM5.2–; morphology identical to CNS tumor	Multidisciplinary review: systemic options discussed; progressive pulmonary disease; neurologically intact
Case 3	79 / M	Left spheno-orbital meningioma, WHO 3 (anaplastic)	Mitotic 25/10 HPF; Ki-67 ≈ 50%; necrosis; cortical-bone invasion; SSTR2+, EMA (focal)+, PR–	Approx. 2 years (from last major resection)	Multiple bilateral pulmonary nodules (largest ≈ 2 cm)	Robotic-assisted bronchoscopy + cone-beam CT; 8 TBNA passes	SSTR2+, EMA (focal)+, PR+, p40–, TTF-1–, synaptophysin–; meningothelial whorls	Refractory craniofacial progression; transitioned to home hospice after multidisciplinary discussion

## Discussion

These three cases illustrate how metastatic propensity reflects an interplay between histologic grade, recurrence burden, and molecular alterations such as NF2 loss or BAP1 inactivation.

Histopathology, immunochemistry, and molecular classification of meningiomas

Meningioma diagnosis has evolved from purely histologic evaluation toward a multidimensional approach that integrates immunohistochemistry and molecular profiling. While histopathologic grading remains the cornerstone of classification, it does not fully capture the biological diversity and recurrence potential of these neoplasms. Recent advances, including DNA methylation-based subclassification and multi-omics integration, have substantially refined prognostic assessment and are now reflected in the 2021 WHO CNS5 framework, which incorporates molecular features such as TERT promoter mutation and CDKN2A/B homozygous deletion as independent grade 3 criteria [6-8]. Histologically, meningiomas are characterized by their meningothelial morphology, including whorl formation, psammoma bodies, and nuclear pseudoinclusions. Despite these classic features, significant morphologic heterogeneity exists across variants with different biological behaviors [9,10]. The WHO grading system remains anchored on three key histologic parameters: mitotic index, brain invasion, and cytologic atypia; supplemented by variant-specific risk categories [11,12]. Ki-67 labeling typically rises with grade: low single digits in grade 1, mid-single to low-double digits in grade 2, and commonly >10% in grade 3 meningiomas [9,13]. Histologic atypia, such as hypercellularity, small-cell change, or necrosis, has been shown to correlate with aggressive molecular subgroups, further underscoring the interplay between morphologic and genetic features [7,14]. Several histologic subtypes of meningioma carry distinct prognostic and diagnostic implications. Chordoid and clear cell meningiomas are categorized as WHO grade 2, while rhabdoid and papillary subtypes are considered WHO grade 3 due to their higher recurrence potential [6,15]. Secretory meningiomas, on the other hand, display a unique immunophenotype, expressing cytokeratins and carcinoembryonic antigen (CEA) within eosinophilic inclusions, whereas metaplastic or lipomatous variants retain meningothelial markers but may histologically mimic sarcomas [16,17]. IHC plays a pivotal role in confirming meningothelial differentiation, identifying variant forms, and serving as a practical surrogate for certain molecular alterations. Standard diagnostic panels typically include epithelial membrane antigen (EMA), vimentin, progesterone receptor (PR), Ki-67, and phosphohistone H3 (PHH3), supplemented by specific markers such as Merlin (for NF2) and BAP1 depending on the morphologic suspicion [9,11,15]. EMA and vimentin are widely expressed in meningiomas and remain the principal lineage markers supporting meningothelial origin [9]. PR positivity is typically seen in lower-grade tumors, and its loss has been associated with higher recurrence and progression risk [17]. Cytokeratin and CEA coexpression is characteristic of secretory meningioma, distinguishing it from metastatic adenocarcinoma [17,18]. Proliferation markers are equally important for grading and prognostication. The Ki-67 labeling index correlates closely with recurrence and overall survival, while PHH3 enhances the reproducibility of mitotic counts, especially in high-grade tumors [19]. IHC also serves as a reliable surrogate for molecular alterations. Loss of Merlin protein expression correlates strongly with NF2 gene inactivation, showing approximately 90% sensitivity and 91% specificity in large series [11]. Similarly, BAP1 loss defines a biologically distinct and clinically aggressive subset of meningiomas characterized by epithelioid morphology, cytokeratin expression, and a specific methylation signature [15,20]. In contrast, high-grade or sarcomatous meningiomas may lose expression of EMA, PR, and vimentin, necessitating molecular confirmation of meningothelial origin [15]. Molecular pathology has fundamentally reshaped the understanding of meningioma biology, bridging morphology and clinical behavior. The most common genomic event remains NF2 gene inactivation associated with chromosome 22q loss, which occurs in roughly half of all sporadic cases and correlates with cytogenetic instability [6]. Non-NF2 mutations, most frequently in TRAF7, KLF4, AKT1, SMO, PIK3CA, POLR2A, SMARCE1, SMARCB1, and PBRM1 define distinct genotype-phenotype subgroups with characteristic locations and histologies, such as the KLF4 + TRAF7 combination associated with secretory meningioma [6,21]. Chromosomal losses involving 1p, 6q, and 14q are strongly linked to higher WHO grades and recurrence risk [6]. Molecular markers now incorporated into the WHO CNS5 classification include TERT promoter mutations and CDKN2A/B deletions, which are sufficient to upgrade a tumor to WHO grade lll even in the absence of high-grade histologic features [6,18]. In addition, BAP1-deficient meningiomas represent a unique molecular class associated with early relapse and may be linked to hereditary tumor syndromes [20]. DNA methylation profiling has emerged as the most accurate molecular classifier, identifying aggressive subsets even within histologically benign tumors [13]. The convergence of histology, immunohistochemistry, and molecular pathology has thus transformed meningioma diagnosis into a multidimensional process. Traditional morphologic evaluation remains essential but is now contextualized within molecular frameworks that refine prognosis and therapeutic decision-making. Emerging evidence supports a comprehensive diagnostic model combining morphologic grade, IHC surrogates such as Merlin and BAP1, and DNA methylation class to improve patient stratification, postoperative surveillance, and selection for adjuvant radiotherapy or clinical trials [6,8,14].

Metastatic meningioma to lung and mediastinum: incidence, latency, risk factors and prognosis

The lung parenchyma represents the most frequent extracranial site of spread, followed by mediastinal lymph nodes, pleura, and, less often, liver or bone involvement [2,3,22-24]. Literature review confirms that the lung remains the predominant metastatic destination, while mediastinal and pleural disease are distinctly uncommon and often accompany disseminated progression [2,25,26]. In one large single-center cohort of 1,193 patients, Ore et al. observed an overall incidence of 0.67%, rising to approximately 2% for WHO grade 2 and 8.6% for grade 3 tumors [3]. Historical compilations and recent pooled analyses yield comparable figures, typically ranging from 0.1% to 0.7% [22,23,25]. These data consistently show that extracranial metastasis, although infrequent, is strongly associated with aggressive histology, high recurrence burden, and prior surgical manipulation.

In most cases, metastatic disease is identified after multiple intracranial recurrences, suggesting that local failure precedes systemic spread [3,24]. The interval from primary diagnosis to metastatic presentation is notably heterogeneous. Low-grade (WHO 1) tumors may metastasize only after protracted clinical courses lasting 10 to 30 years, particularly in the context of repeated recurrence or incomplete resection [27]. In contrast, anaplastic (WHO 3) meningiomas tend to metastasize rapidly, sometimes within months to a few years of diagnosis [28]. Case reports describe pulmonary metastases arising more than two decades after resection of grade I primaries, highlighting the potential for latency even among histologically benign lesions. Conversely, high-grade tumors with aggressive molecular features can disseminate early, frequently within two to three years, and carry a correspondingly poorer prognosis [2,24,29].

From a clinicopathologic perspective, thoracic metastases manifest a broad morphological spectrum that reflects both the primary tumor’s histology and its molecular evolution. Pulmonary lesions may appear as solitary nodules or as multiple, randomly distributed nodules that mimic metastatic carcinoma on imaging [25,30]. Mediastinal lymph node involvement, while less common, has been biopsy-confirmed in case series [31]. Pleural metastases and effusions are rare but reported in disseminated disease; such patterns indicate that hematogenous spread predominates, although lymphatic extension via dural or venous sinus invasion has also been proposed as a mechanism of dissemination disease [26]. The most consistent predictor of extracranial spread is tumor grade. Higher-grade (WHO 2 and 3) meningiomas metastasize earlier and more frequently than grade I lesions. Additional risk factors include repeated recurrence, prior radiotherapy, and incomplete resection, all of which may increase hematogenous access and tumor adaptation. Histologic subtypes such as rhabdoid, papillary, or clear cell meningiomas, although sometimes categorized as grade 1 or 2, tend to behave more aggressively and have been overrepresented among metastatic cases [30,32]. BAP1 loss has emerged as a key molecular marker of aggressive potential and may drive metastasis even in histologically low-grade tumors [20,33]. Additional structural or anatomical factors such as dural sinus involvement, skull-base or bone invasion, it also may further facilitate hematogenous spread by providing venous channels for tumor embolization [2,34]. Genomic analyses of metastatic meningiomas support a model of stepwise molecular evolution. Longitudinal sequencing of matched primary and lung metastatic specimens has confirmed early NF2 loss with subsequent accumulation of alterations in BRCA2, SETD2, ATM, and Beta-2-microglobulin, along with increased tumor mutational burden, implying progressive genomic instability and immune-evasion mechanisms [35,36]. Such molecular heterogeneity likely underlies the wide variability in latency and clinical course seen across reported cases.

Clinically, most pulmonary or mediastinal metastases are discovered incidentally during follow-up imaging, though some patients present with cough, dyspnea, or pleuritic chest pain in advanced stages. Diagnostic work-up typically involves chest CT or whole-body PET/CT to identify metabolically active lesions, followed by tissue confirmation via robotic bronchoscopy, CT-guided or surgical biopsy [3,30,31]. Immunohistochemical comparison with the original CNS specimen remains critical for definitive diagnosis and to distinguish metastatic meningioma from rare primary pulmonary meningioma or other spindle-cell neoplasms. Surgical metastasectomy provides durable control in selected oligometastatic, indolent cases; radiotherapy and systemic agents are used for palliation in multifocal or unresectable disease [30,32]. Thoracic radiotherapy can provide local palliation in unresectable disease. Systemic therapy results are inconsistent; cytotoxic chemotherapy yields modest benefit at best, whereas targeted agents such as sunitinib or anti-VEGF combinations and checkpoint inhibitors have produced variable or short-lived responses in limited series [33,36]. Reported survival outcomes depend on grade and burden of disease: patients with high-grade or multifocal metastases experience rapid progression and shortened survival, while selected patients with low-grade, slowly progressive lesions can remain stable for years with local management [2,24]. For example, Cui et al. described a patient with a grade I petrous apex meningioma who, after two incomplete resections and adjuvant radiotherapy, remained stable despite slow progression of pulmonary metastases [29]. Similarly, Hanna et al. reported a grade I case with metastasis emerging several years after initial surgery and radiation, illustrating how indolent histology does not preclude eventual systemic spread [37]. Based on available evidence, patients with high-grade disease, aggressive histologic variants, multiple recurrences, venous invasion, or high-risk molecular alterations (e.g., BAP1 loss) may warrant consideration of periodic thoracic imaging, although this remains a suggestion given the rarity of metastatic cases.

Case 1 highlights long-latency metastasis in a low-grade but molecularly high-risk tumor; Case 2 demonstrates rapid dissemination in an anaplastic tumor; and Case 3 shows progressive extracranial spread in a highly recurrent WHO grade 3 meningioma influenced by treatment morbidity.

## Conclusions

Thoracic dissemination of meningiomas is uncommon but clinically consequential, with the lung parenchyma as the predominant extracranial site, and mediastinal or pleural involvement occurring less frequently. The three cases presented illustrate distinct metastatic trajectories: long-latency spread from a recurrent, BAP1-altered tumor; rapid dissemination from an anaplastic (WHO grade 3) lesion; and progression in a multiply operated skull-base tumor complicated by treatment morbidity. Across these scenarios, morphologic grade alone did not fully predict metastatic risk. Instead, recurrence history, surgical with venous sinus involvement, and molecular features, particularly BAP1 loss, better captured the aggressive potential. These observations support a risk-adapted surveillance strategy in which patients with high-grade, aggressive histology, extensive recurrence burden, or known molecular risk (BAP1 loss, CDKN2A/B deletion, TERT promoter mutation) may warrant selective chest imaging in addition to routine neuroimaging. Pathologic confirmation of extracranial lesions remains essential, using lineage-concordant IHC (EMA, SSTR2, PR) and, when needed, molecular correlation with the index intracranial tumor. Treatment of metastatic meningioma is frequently palliative; local approaches (metastasectomy or focal radiotherapy) can provide durable control for oligometastatic disease, whereas systemic options (anti-VEGF, mTOR inhibition, checkpoint blockade) show variable activity and should preferentially be delivered within clinical trials. Given the rarity of thoracic spread, carefully documented case series remain valuable for clarifying prognosis, refining surveillance algorithms, and informing future therapeutic studies aimed at this high-risk subset.

## References

[1] Wang JZ, Landry AP, Raleigh DR (2024). Meningioma: International Consortium on Meningiomas consensus review on scientific advances and treatment paradigms for clinicians, researchers, and patients. Neuro Oncol.

[2] Himič V, Burman RJ, Fountain DM, Hofer M, Livermore LJ, Jeyaretna DS (2023). Metastatic meningioma: a case series and systematic review. Acta Neurochir (Wien).

[3] Ore CL, Magill ST, Yen AJ (2020). Meningioma metastases: incidence and proposed screening paradigm. J Neurosurg.

[4] Horbinski C, Berger T, Packer RJ, Wen PY (2022). Clinical implications of the 2021 edition of the WHO classification of central nervous system tumours. Nat Rev Neurol.

[5] Lotsch C, Warta R, Herold-Mende C (2024). The molecular and immunological landscape of meningiomas. Int J Mol Sci.

[6] Deng J, Hua L, Bian L (2022). Molecular diagnosis and treatment of meningiomas: an expert consensus (2022). Chin Med J (Engl).

[7] Maas SL, Stichel D, Hielscher T (2021). Integrated molecular-morphologic meningioma classification: a multicenter retrospective analysis, retrospectively and prospectively validated. J Clin Oncol.

[8] Trybula SJ, Youngblood MW, Karras CL (2024). The evolving classification of meningiomas: integration of molecular discoveries to inform patient care. Cancers (Basel).

[9] Kolles H, Niedermayer I, Schmitt C (1995). Triple approach for diagnosis and grading of meningiomas: histology, morphometry of Ki-67/Feulgen stainings, and cytogenetics. Acta Neurochir (Wien).

[10] Mawrin C, Perry A (2010). Pathological classification and molecular genetics of meningiomas. J Neurooncol.

[11] Kashiwagi-Hakozaki M, Ikemura M, Miyawaki S, Teranishi Y, Okano A, Saito N, Ushiku T (2025). Merlin immunohistochemistry is a reliable surrogate marker for NF2 gene alterations in meningioma. Histopathology.

[12] Maier AD, Bartek J Jr, Eriksson F, Ugleholdt H, Juhler M, Broholm H, Mathiesen TI (2020). Clinical and histopathological predictors of outcome in malignant meningioma. Neurosurg Rev.

[13] Sahm F, Scrimpf D, Stichel D (2017). DNA methylation-based classification and grading system for meningioma: a multicentre, retrospective analysis,. Lancet Oncol.

[14] Nassiri F, Liu J, Patil V (2021). A clinically applicable integrative molecular classification of meningiomas. Nature.

[15] Lucas CG, Devine P, Solomon DA (2021). Sarcomatous meningioma: diagnostic pitfalls and the utility of molecular testing. J Neuropathol Exp Neurol.

[16] Hanna C, Matthew W, Dwayne C (2023). Review of meningioma diagnosis and management. Egypt J Neurosurg.

[17] Rocchetti JR, Haldar N, Haldar D, Andrews DW, Werner-Wasik M (2025). Recurrent grade I parafalcine meningioma: management challenges and genomic insights. Cureus.

[18] Patel Z, Wang JZ, Merali Z (2023). DNA methylation profiling of a lipomatous meningioma: illustrative case. J Neurosurg Case Lessons.

[19] Congivaram H, Thirunavu V, Santana-Santos L (2024). Path-59. Histologic correlates for molecular features in meningiomas. Neuro Oncol.

[20] Drabent P, Touat M, Benusiglio PR (2025). A specific methylation class identifies BAP1-deficient meningiomas, including meningeal tumours with poorly differentiated non-rhabdoid histology. Neuropathol Appl Neurobiol.

[21] Wiemels J, Wrensch M, Claus EB (2010). Epidemiology and etiology of meningioma. J Neurooncol.

[22] Stoller JK, Kavuru M, Mehta AC, Weinstein CE, Estes ML, Gephardt GN (1987). Intracranial meningioma metastatic to the lung. Cleve Clin J Med.

[23] Bazer D, Zabrocka E, Torres M, Frontario A, Kowalska A (2022). NCMP-11. Metastatic meningioma: an unusual course of a common disease. Neuro Oncol.

[24] Rhim JK, Sheen SH, Noh JS, Chung BS (2004). Pulmonary metastasis of malignant meningioma. J Korean Neuro Soc.

[25] Watanabe G, Young K, Rauber E (2024). A systematic review of extraneural meningioma metastasis: timing, evolution and outlook. J Neurooncol.

[26] Wu L, Luo S (2024). A case report of Extra-neurologic metastasis of ventricular meningioma with literature review. Radiol Case Rep.

[27] Di Lorenzo S, Farese S, Balbo C (2025). The first case report of a solitary pulmonary metastasis of a transitional meningioma and literature review [PREPRINT]. Int J Mol Sci.

[28] Huntoon K, Toland AM, Dahiya S (2020). Meningioma: a review of clinicopathological and molecular aspects. Front Oncol.

[29] Cui J, Zou X, Han Y, Jiang J (2024). Benign extracranial meningioma with pulmonary metastasis: a case report and review of literature. J Med Case Rep.

[30] Asioli S, Senetta R, Maldi E, D'Ambrosio E, Satolli MA, Bussolati G, Cassoni P (2007). "Benign" metastatic meningioma: clinico-pathological analysis of one case metastasising to the lung and overview on the concepts of either primitive or metastatic meningiomas of the lung. Virchows Arch.

[31] Sungkaro K, Sae-heng S (2022). Metastasis of malignant intracranial meningioma to the lung: report of a case and review of the relevant literature. Iran J Neurosurg.

[32] Kleynberg RL, Kleynberg LM, Kleynberg VM (2013). Benign or malignant? Extensive pulmonary metastasis of an intracranial meningioma—unique radiographic and histopathologic features. Int J Case Rep Image.

[33] Shankar GM, Santagata S (2017). BAP1 mutations in high-grade meningioma: implications for patient care. Neuro Oncol.

[34] Ahmed N, Chaurasia B (2025). Deciphering extracranial metastasis in high-grade meningiomas: insights from a case study and literature review. Ann Med Surg (Lond).

[35] Cosgrove N, Fitzpatrick OM, Grogan L, Hennessy BT, Furney SJ, Toomey S (2024). Case report: clonal evolution analysis of a rare case of meningioma lung metastases identifies actionable alterations in matched longitudinal tumour samples. Front Oncol.

[36] Cieslak E, Kepka L, Fijuth J (2001). Metastases of intracranial meningioma with sarcomatous change to the lung: a case report. Nowotwory.

[37] Hanna R, Feldman AM, Keller CE, Siddiqui MS (2020). A grade I intracranial meningioma with metastasis to multiple vertebral bodies: a case report and literature review. Cureus.

